# Two-dimensional van der Waals C_60_ molecular crystal

**DOI:** 10.1038/srep12221

**Published:** 2015-07-17

**Authors:** C. D. Reddy, Zhi Gen Yu, Yong-Wei Zhang

**Affiliations:** 1 Institute of High Performance Computing, A*STAR, Singapore 138632

## Abstract

Two-dimensional (2D) atomic crystals, such as graphene and transition metal dichalcogenides *et al.* have drawn extraordinary attention recently. For these 2D materials, atoms within their monolayer are covalently bonded. An interesting question arises: Can molecules form a 2D monolayer crystal via van der Waals interactions? Here, we first study the structural stability of a free-standing infinite C_60_ molecular monolayer using molecular dynamic simulations, and find that the monolayer is stable up to 600 K. We further study the mechanical properties of the monolayer, and find that the elastic modulus, ultimate tensile stress and failure strain are 55–100 GPa, 90–155 MPa, and 1.5–2.3%, respectively, depending on the stretching orientation. The monolayer fails due to shearing and cavitation under uniaxial tensile loading. The highest occupied molecular orbital (HOMO) and lowest unoccupied molecular orbital (LUMO) of the monolayer are found to be delocalized and as a result, the band gap is reduced to only 60% of the isolated C_60_ molecule. Interestingly, this band gap can be tuned up to ±30% using strain engineering. Owing to its thermal stability, low density, strain-tunable semi-conducting characteristics and large bending flexibility, this van der Waals molecular monolayer crystal presents aplenty opportunities for developing novel applications in nanoelectronics.

Two-dimensional (2D) atomic materials, such as graphene, hexagonal boron nitride, silicene, phosphorene, transition metal dichalcogenides and layered oxides, have drawn considerable attention recently due to their unique crystalline structures and fascinating properties^1^,^2^,^3^,^4^,^5^. In these 2D atomic materials, covalent bonds make them strong within their monolayers; while relatively weak van der Waals (vdW) forces across these monolayers enable great flexibility for design and assembly. To fully utilize their various properties in different 2D atomic materials, it was proposed to stack different 2D materials via van der Waals forces to achieve extraordinary properties and novel functionalities^5^,^6^.

On the other hand, in molecular solids, molecules are also held together in bulk form by weak vdW forces^7^. These solids are of low density and low stiffness compared to their atomic counterparts^7^,^8^. Remarkably, the 2D form of molecular solids has been synthesised through self-assembly on various substrates^9^,^10^,^11^,^12^,^13^,^14^,^15^. It is noted that the self-assembly of the vdW molecular crystals on substrate depends on many factors, such as surface properties, adhesion energies, molecular size and shape, orientations, pressure, temperature and solvents used to disperse the molecules^9^,^10^,^11^,^12^,^13^. Currently, forming a well-defined free-standing molecular monolayer remains a daunting task due to the weak binding forces between its constituent molecules^9^.

Recently, 2D organic molecular crystals have been synthesized and several interesting applications in novel devices have been demonstrated^14^,^15^,^16^,^17^,^18^,^19^,^20^,^21^. It is noted that the C_60_ molecules have been used as a basic building block in many recent applications, for example, in organic photovoltaics^14^,^16^, molecular electronics^15^,^17^, cosmetics and healthcare^18^,^19^,^20^,^21^,^22^,^23^. Although the C_60_ molecules have been self-assembled into a stable monolayer thin film on substrates,^11^,^14^ the existence of the free-standing van der Waals molecular monolayer crystal, that is, without the support of a substrate, remains unknown. Before the seminal work by Geim and co-workers^24^, it was commonly believed that free-standing 2D atomic materials are thermodynamically unstable. Their work, however, proved the existence of the free-standing monolayer atomic crystal and thus busted the previous belief^24^. Therefore, a few interesting questions arise: Can the C_60_ molecules form a stable free-standing 2D molecular monolayer crystal? If the answer is yes, then, up to what temperature? What are the mechanical and failure behaviour of the monolayer? What are its electronic characteristics? Clearly, answers to these questions are not only of scientific significance but also of technological impact. Thus, studying the stability of the C_60_ molecular monolayer and revealing their mechanical and electronic properties constitute the subject of the present study.

## Results and Discussions

### Structure

In the crystalline C_60_ solid, the C_60_ molecules adhere together by weak vdW forces and exhibit the face-centered cubic (FCC) structure at room temperature^25^. Experimental evidence shows that these molecules are able to self-assemble into a 2D monolayer on a substrate by adopting the closed pack hexagonal structure^13^,^16^. The nearest neighbour distance between the C_60_ molecules in the hexagonal structure was reported to be in the range of 9.5 to 10 Å^26^,^27^.

**Figure 1:**
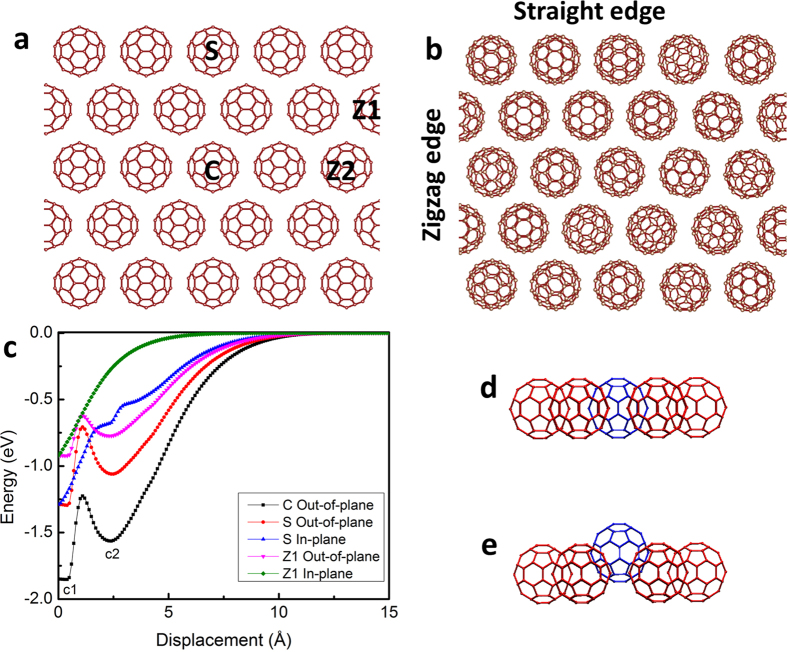
The C_60_ molecules arranged in a hexagonal pattern with the same orientation (**a**) and energetically optimised structure after random perturbations (**b**). Three different molecular positions, centre (C), straight edge (S) and zigzag edge (Z1) are considered to study the interaction energy versus the displacement from the equilibrium position of a C_60_ molecule (**c**). In case of the vertical (out-of-plane) pulling, there are two energy minimal positions (c1) and (c2) and their corresponding structures are shown in (**d**) and (**e**). In horizontal (in-plane) pulling case, there is one energy minimum exists which corresponds to equilibrium position.

First, we examine the stability of the free-standing C_60_ molecular monolayer crystal. All C_60_ molecules are arranged in a perfect hexagonal pattern as shown in Fig. 1a. These molecules are held together by van der Waals forces. Our calculations show that the vdW interactions are strong enough to enforce a stable in-plane monolayer structure in absence of temperature. In presence of temperature, however, we find that each C_60_ molecule performs random thermal fluctuations in all the degrees of freedom, including both translational and rotational ones. After thermal equilibration and subsequent energy minimization, the C_60_ molecules adopt a hexagonal lattice structure, but their orientations are randomly distributed (Fig. 1b).

### Binding energy

Energy analysis is performed to estimate the binding strength of the monolayer structure. The energy required to pull a C_60_ molecule in the monolayer from its equilibrium position depends on the coordination number as well as the pulling direction. The coordination number for a C_60_ at the zigzag edge is either 3 (for Z1 molecule) or 5 (for Z2 molecule), depending on the location of the molecule on the edge. The coordination number is 4 and 6 for a C_60_ at the straight edge (S) and in the centre (C), respectively (Fig. 1a,b). The interaction energy is measured by displacing the molecule in the prescribed direction (in-plane or out-of-plane) while keeping all other molecules fixed at their original positions. Figure 1c shows the variation of interaction energy with the pulling displacement of the molecule from its equilibrium position. Among the five curves in Fig. 1c, three are under the out-of-plane pulling and the other two are under the in-plane pulling. It is seen that all the three out-of-plane pulling curves show the same trend and have two minima, with the left one (at position c1) being the equilibrium position (Fig. 1d) and the right one (at position c2) being the meta-stable equilibrium position with 2.5 Å away from the equilibrium position (Fig. 1e). However, the other two curves that belong to the in-plane pulling show the same trend as the van der Waals interaction energy between any two molecules/atoms. From Fig. 1c, it is clear that in a finite C_60_ monolayer, the C_60_ molecules at the zigzag edge are weakly bonded to their surrounding molecules. The binding energy of the C_60_ molecule in the infinite 2D monolayer is found to be 0.85 eV, which is half of that in the C_60_ FCC crystal^28^,^29^.

### Thermal stability

**Figure 2:**
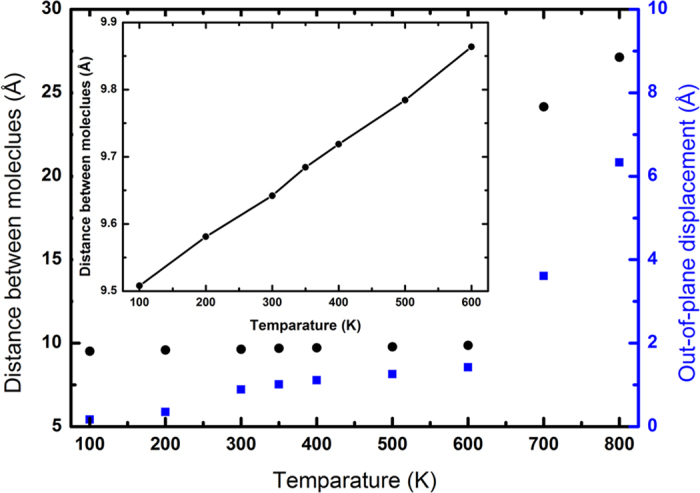
The variation of the average intermolecular distance (black) and the average out-of-plane distance (blue) of the molecules from its equilibrium with temperature. The change in the average intermolecular distance and the average out-of-plane distance are very small until 600 K and all the molecules are adhere with their neighbours. Beyond 600 K, the C_60_ molecules vibrate violently, which destroys the crystal structure. The Inset shows the linear variation of the average intermolecular distance with temperature below 600 K.

In the following, we examine the thermal stability of infinitely large free-standing C_60_ molecular monolayer by using periodic boundary conditions. All molecules in the system are equilibrated at prescribed temperatures for 10 ns. During the equilibration, the molecules are vibrating around their equilibrium positions. We calculate the out-of-plane distance from its equilibrium positions as shown in Fig. 2. It is seen that the average out-of-plane distance of all the molecules increases slowly with temperature up to 600 K, but beyond this, the distance increases sharply, indicating the loss of stability (Fig. 2). After the loss of the stability, some of the molecules aggregated into three dimensional clusters while others fly away. Figure 2 also shows the variation of the average inter-molecular distance with temperature from which we also calculated the coefficient of thermal expansion for the monolayer and found that it is about 8 × 10^−5^ K^−1^, which is in the same order of most of solid structures^30^. We also found that the finite C_60_ monolayer is stable up to 150 K, and beyond this temperature, the molecules at the zigzag edge become unstable and the finite structure evolves into a 3D aggregate as shown in Fig. S1 in Supplementary material. It is noted that the thermal degradation temperature of the solid 3D crystal was experimentally measured and reported to be in the range of 970 ~ 1300 K^31^,^32^,^33^, which nearly doubles the destabilization temperature of the 2D monolayer.

### Bending stability

In order to demonstrate that the 2D C_60_ monolayer is able to sustain bending, we have constructed different sizes of C_60_ monolayer nanotubes and tested for their structure stability (under periodic condition along the nanotube axis). At room temperature, we find that the nanotubes with a diameter greater than 90 Å (the curvature below 0.022 Å^−1^) are stable. For nanotubes with a curvature greater than 0.022 Å^−1^, we find that thermal vibrations of C_60_ molecules destabilize the nanotubes and transform the C_60_ nanotubes into C_60_ nanowires at room temperature. We have added the C_60_ nanotube and nanowire structures in the Supplementary material Fig. S2.

### Stress-strain relation and failure

**Figure 3:**
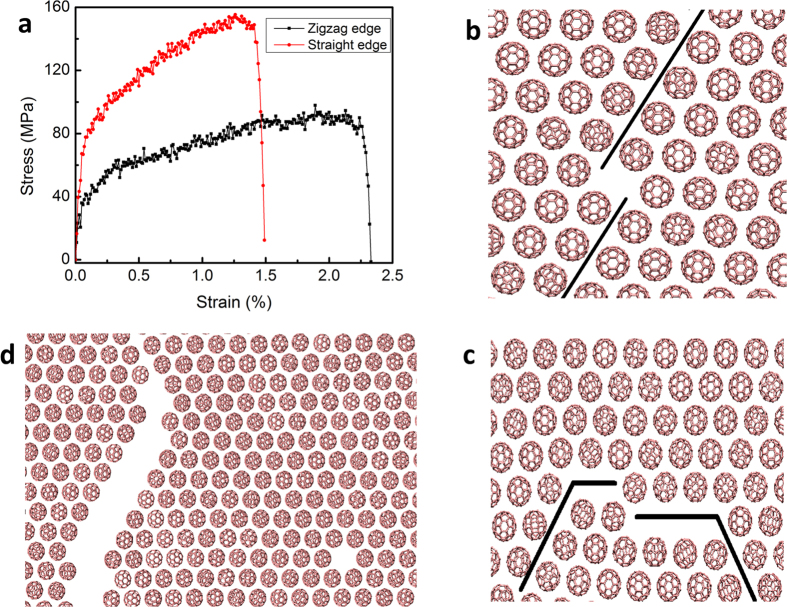
The stress-strain curves of the monolayer under uniaxial tensile loading along the zigzag and straight edge directions (**a**). (**b**) and (**c**) show the failure patterns under the uniaxial tensile strain along the zigzag and straight edge directions, respectively. Note that shear deformation along the 60º angle with respect to the horizontal axis creates voids in the structure (**b** and **c**). (**d**) shows a fully developed crack, the healing of some of the shear lines and the formation of stable voids.

To investigate the elastic properties of the molecular monolayer, we apply uniaxial tensile strain and measure the tensile stress of the system. Figure 3 shows the stress-strain curves under uniaxial loading along the zigzag and straight edge directions, which indicate that the system behaves like a nonlinear material. The stress in the system rises sharply for smaller strains, then increases slowly when increasing the applied strain further, and drops drastically. The monolayer behaves like an elastic-plastic material. The transition is attributed to the rotation of the molecules, which causes the slope to change from the sharp to slow increase parts of the stress-strain curve. The elastic modulus is estimated to be 55 GPa for the zigzag direction and 100 GPa for the straight edge directions, respectively. The ultimate tensile stress is calculated to be 90 MPa and 155 MPa in the zigzag and straight edge directions, respectively. Due to the anisotropic arrangement of the molecules in the monolayer, the elastic modulus and failure strength are different in the zigzag and straight directions. In addition, the failure strains are also different with 2.3 and 1.5% being in the zigzag and straight edge directions. Before the rupture, all the molecules adhere with neighbours. The failure starts to occur when part of molecules slides against another part along the 60° line (the black line in Fig. 3b,c), leading to the void formation in the structure. Beyond the failure strain, some of the shear lines heal, some form stable voids, while some propagate and lead to the crack growth as shown in Fig. 3d. Interestingly, such voids and cracks have been observed in C_60_ monolayer on graphene/SiC substrate^16^.

### Band gap calculations

**Figure 4:**
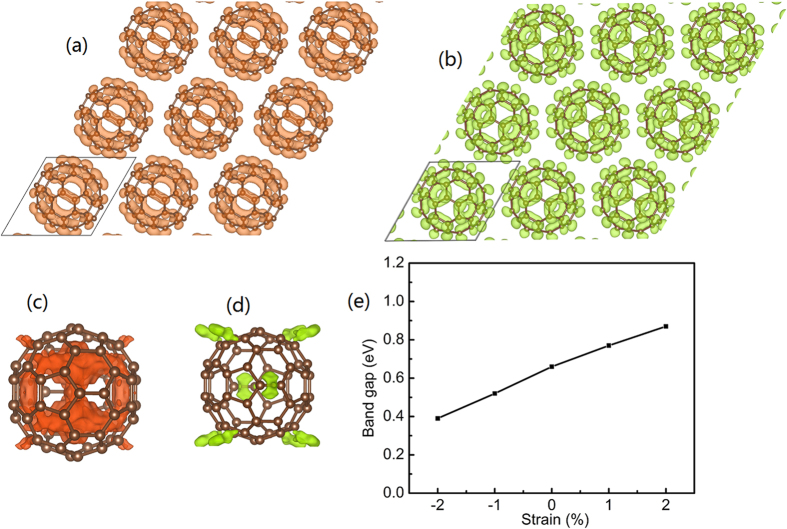
Isosurfaces of band-decomposed charge densities at the top of the valence band (HOMO) and the bottom of the conduction band (LUMO) of the C_60_ monolayer (**a**) and (**b**), respectively. The HOMO, and LUMO of isolated C_60_ molecule are shown in (**c**) and (**d**), respectively. The variation of calculated band gap with biaxial strain for the C_60_ monolayer (**e**). The isovalue of 0.03  eV/Å^3^ is used.

Based on the optimized lattice constants and atomic coordinates obtained by using molecule dynamics calculations, we build a super cell of the 2D monolayer with an inter molecular distance of 9.50 Å as shown in Fig. 4a. We calculate the highest occupied molecular orbital (HOMO) and the lowest unoccupied molecular orbital (LUMO) and the results are shown in Fig. 4(a,b). For the C_60_ monolayer, it is found that the HOMO and LUMO cover the carbon-carbon bonds, which may act as an electron transport pathway and result in high electron mobility. For comparison, we also investigate the HOMO and LUMO of an isolated C_60_ fullerene. It is found that the HOMO and LUMO of the isolated C_60_ fullerene are located between the carbon-carbon bonds and are highly localized. The calculated band gap of the C_60_ monolayer and isolated C_60_ molecule are 0.66 eV and 1.63 eV, respectively. These results are consistent with the understanding that localized HOMO and LUMO may result in a large band gap, while delocalized HOMO and LUMO may result in a small band gap. In addition, the calculated band gap of the isolated C_60_ molecule is consistent with the previously reported value of 1.7–1.9 eV^34^.

We further explore the strain engineering on the band gap of the C_60_ monolayer by applying biaxial strain in the range from −2% to 2%. The negative values represent the compressive strain and the positive values represent the tensile strain. Figure 4(e) shows the results of the calculated band gap as a function of the applied strain. It is seen that the band gap has a nearly linear function with the strain. Under the compressive strain, the band gap decreases with increasing strain; while under the tensile strain, the band gap increases with increasing strain. Remarkably, the band gap can be tuned in the range of ±30% by the strain engineering (Fig.4e).

In the present study, we have computationally shown the existence of stable C_60_ molecular monolayer. Due to its thermal stability, low density, strain-tunable semiconducting characteristics, moderate elastic modulus and high bending flexibility, it is envisioned that this monolayer material can be used in many novel applications. For example, this monolayer may be used for storage devices for a wide range of molecules in bio- and energy applications^20^,^35^. In addition, due to the rotational degrees of freedom of the molecules in the monolayer, it may also be used as a highly efficient solid lubricant between moving parts in nanodevices^36^. Because of the 2D arrangement of the molecules and tunable semi-conducting characteristics, this layer can be used as a standalone device or be part of 2D heterogeneous structural devices^11^,^15^,^16^.

### 

**In summary**, we have computationally investigated the structure, mechanical and electronic properties of the free-standing van der Waals monolayer crystal using C_60_ molecules. Using molecular dynamics simulations, we have shown that the monolayer infinite structure is stable up to 600 K; the thermal expansion coefficient is about 8 × 10^−5^ K^−1^; and the stress-strain curve of the monolayer follows an approximately bilinear behaviour. Due to the molecular anisotropic arrangement, the failure strain is orientation-dependent with 1.5 and 2.3% being in the zigzag and straight directions, respectively. Density functional simulations were used to calculate the HOMO and LUMO of the C_60_ monolayer and an isolated C_60_ molecule. It is found that the monolayer exhibits a band gap of 0.6 eV, which is much lower than that of isolated C_60_ molecular band gap of 1.6 eV. The band gap can be tuned by strain engineering from 0.4 to 0.8 eV (±30%). This molecular monolayer is expected to find aplenty opportunities in the future electronic, biomedical and energy storage applications.

## Methods

In order to study the structure and stability of the C_60_ monolayer crystal, C_60_ molecules are arranged in a hexagonal network as shown in Fig. 1. Large-scale Atomic/Molecular Massively Parallel Simulator (LAMMPS) molecular dynamics simulation package along with Adaptive Intermolecular Reactive Empirical Bond Order (AIREBO) potential is used to simulate the covalent and vdW interactions of the molecular crystal structure^37^,^38^. To mimic the infinite structure in two-dimensions, in-plane periodic boundary conditions are used. The static calculations are used to find the interaction energies when the molecule displace from its equilibrium position in a desired direction and kept all neighbours at their original positions. For thermal stability studies, NVE ensemble is used to equilibrate the system at desired temperatures. To calculate the stress in the system uniform incremental tensile strain applied under NPT ensemble. First-principles calculations are carried out in the framework of density functional theory (DFT) as implemented in the Vienna ab initio simulation package (VASP)^39^,^40^. The generalized gradient approximation (GGA) within Perdew-Burke-Ernzerhof (PBE) formalism is employed for the exchange-correlation potential^41^. The projector augmented wave (PAW) method^42^ and a plane-wave basis set with an energy cutoff of 500 eV are used in the calculations. For geometry optimizations, the Brillouin-zone integration is performed using a regular 3 × 3 × 1 k mesh within the Monkhorst-Pack scheme. The electronic density of states calculations of the Brillouin zone is sampled by employing a k-point grid of 5 × 5 × 1. The Gaussian smearing method is employed and the width of the smearing is chosen as 0.01 eV in all relaxations. The convergence criterion of the self-consistent field calculations is set to 10^−5^ eV for the total energy. To prevent spurious interaction between isolated C_60_, a large vacuum spacing (at least 15 Å) is introduced. By using the conjugate gradient method, we optimize the atomic positions and lattice constants until the atomic forces are less than 0.01 eV/Å.

## Additional Information

**How to cite this article**: Reddy, C.D. *et al.* Two-dimensional van der Waals C^60^ molecular crystal. *Sci. Rep.* **5**, 12221; doi: 10.1038/srep12221 (2015).

## Supplementary Material

Supplementary Information
