# Supplementary material for: Two-dimensional van der Waals C60 molecular crystal
Source: Sci Rep. 2015 Jul 17;5:12221. doi: 10.1038/srep12221 (PMC4505331; doi:10.1038/srep12221)
Supplement: Supplementary Information [file srep12221-s1.pdf]

Supplementary material:

Two-dimensional van der Waals C_{60} molecular crystal

C.D. Reddy*, Zhi Gen Yu & Yong-Wei Zhang*

Institute of High Performance Computing, A*STAR, Singapore 138632

*Correspondence and requests for materials should be addressed to C.D.R. (reddy@ihpc.a-star.edu.sg) or Y.W.Z. (zhangyw@ihpc.a-star.edu.sg)

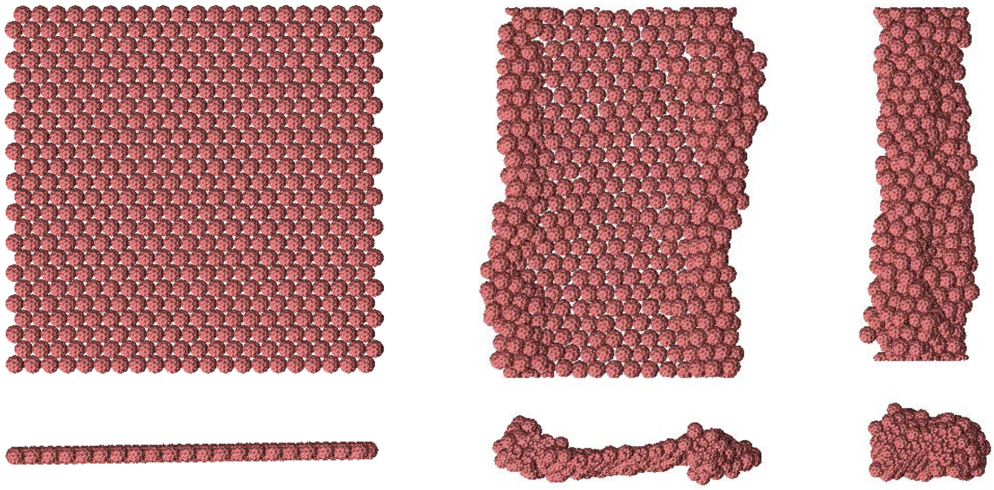

Figure S1: Snapshots of finite 2D C_{60} monolayer at room temperature. The left panels (both the top and side views) show the initial structure, the middle panels show unstable zigzag edges, and the right panels show the formation of a nanowire. In the calculations, we used periodic boundary along the straight edge direction and free edge condition along the zigzag direction.

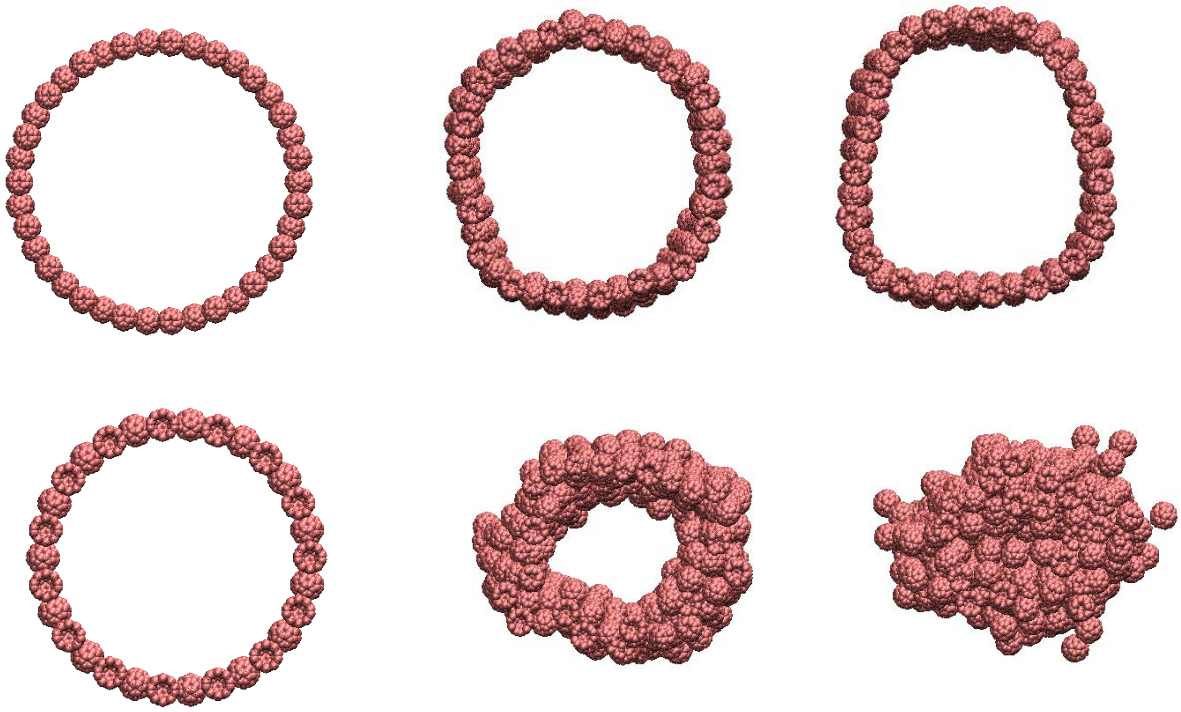

Figure S2: Snapshots of C_{60} nanotubes of two different diameters under equilibrium at 300 K. Top row: The nanotube with a diameter of 95 Å is stable and retains the tube shape. Bottom row: The nanotube of a diameter of 80 Å is unstable and destroyed by thermal fluctuations. Periodic boundary condition is used along the tube axis in the simulations.
